# IMA Genome - F16: Draft genome assemblies of *Fusarium marasasianum*, *Huntiella abstrusa*, two *Immersiporthe knoxdaviesiana* isolates, *Macrophomina pseudophaseolina*, *Macrophomina phaseolina*, *Naganishia randhawae*, and *Pseudocercospora cruenta*

**DOI:** 10.1186/s43008-022-00089-z

**Published:** 2022-02-23

**Authors:** Brenda D. Wingfield, Lieschen De Vos, Andi M. Wilson, Tuan A. Duong, Niloofar Vaghefi, Angela Botes, Ravindra Nath Kharwar, Ramesh Chand, Barsha Poudel, Habibu Aliyu, Martin J. Barbetti, ShuaiFei Chen, Pieter de Maayer, FeiFei Liu, Sudhir Navathe, Shagun Sinha, Emma T. Steenkamp, Hiroyuki Suzuki, Kalonji A. Tshisekedi, Magriet A. van der Nest, Michael J. Wingfield

**Affiliations:** 1 Department of Biochemistry, Genetics and Microbiology, Forestry and Agricultural Biotechnology Institute, University of Pretoria, Pretoria, 0028 South Africa; 2 Centre for Crop Health, University of Southern Queensland, Toowoomba, Australia; 3 School of Molecular and Cell Biology, University of the Witwatersrand, Johannesburg, South Africa; 4 Center of Advanced Study in Botany, Institute of Science, Banaras Hindu University, Varanasi, India; 5 Department of Mycology and Plant Pathology, Institute of Agricultural Sciences, Banaras Hindu University, Varanasi, India; 6 Institute of Process Engineering in Life Science, Karlsruhe Institute of Technology, Karlsruhe, Germany; 7 School of Agriculture and Environment and the UWA Institute of Agriculture, University of Western Australia, Perth, Australia; 8 China Eucalypt Research Centre, Chinese Academy of Forestry, Zhanjiang, Guangdong Province China; 9 Agharkar Research Institute, Pune, Maharashtra India; 10 Biotechnology Platform, Agricultural Research Council, Pretoria, South Africa

## IMA GENOME-F 16A

### Draft genome assembly of *Fusarium marasasianum*

#### Introduction

Many plants are thought to have at least one *Fusarium*-associated disease with more than 80% of economically important plants affected by at least one *Fusarium* disease (Leslie and Summerell 2006). The socioeconomic importance of *Fusarium* is particularly evident when considering the *Fusarium fujikuroi* species complex (FFSC, sensu Geiser et al. 2021). This monophyletic group contains 65 species and numerous cryptic species (Yilmaz et al. 2021). More than 50 species in the FFSC have publicly available genomes (www.ncbi.nlm.nih.gov), indicative of their economic importance.

A number of recent studies showed that the FFSC complex contains four large clades (Herron et al. 2015; Sandoval-Denis et al. 2018; Yilmaz et al. 2021). One of these corresponds to the so-called “American” clade that was initially proposed to reflect the biogeography of the species it contains (O’Donnell et al. 1998). For example, *Fusarium circinatum*, the pine pitch canker pathogen, is thought to be native to Mexico and Central America (Drenkhan et al. 2020), where it likely co-evolved with its *Pinus* hosts (Herron et al. 2015; O’Donnell et al. 1998; Wikler and Gordon 2000). The American clade also includes five additional species associated with *Pinus* species in Colombia. These species are *F. fracticaudum, F. pininemorale, F. parvisorum, F. marasasianum,* and *F. sororula*, of which *F. parvisorum, F. marasasianum,* and *F. sororula* displayed levels of pathogenicity that were comparable to those of *F. circinatum* on susceptible *Pinus* species (Herron et al. 2015).

The risk that the various American clade species pose to forestry in Colombia and globally has provided the impetus for projects aiming to sequence their genomes. To complement the genomic resources available for *F. circinatum* (Fulton et al. 2020; van der Nest et al. 2014a; Van Wyk et al. 2018; Wingfield et al. 2012, 2018a), the genomes of *F. pininemorale* (Wingfield et al. 2017), *F. fracticaudum* (Wingfield et al. 2018b) and *F. sororula* (van der Nest et al. 2021) have been published. Here we present the whole genome sequence for the pine pathogen *F. marasasianum*, named after the late South African professor Walter “Wally” F.O. Marasas (Wingfield and Crous 2012) who specialised in the taxonomy of *Fusarium* species and their associated mycotoxins.

#### Sequenced strain

**Colombia**: Volconda, Valle del Cauca, 4.0297222° N, 76.4183334° W, isolated from *Pinus tecunumanii,* 2005, *Carlos A Rodas* (CMW 25512; PREM 63311-dried culture) (Herron et al. 2015).

#### Nucleotide sequence accession number

This Whole Genome Shotgun project has been deposited at DDBJ/ENA/GenBank under the accession JAJEQZ000000000. The version described in this paper is version JAJEQZ010000000.

#### Materials and methods

*Fusarium marasasianum* CMW 25512 was grown on ½ potato dextrose agar (PDA) medium consisting of 20% w/v PDA and 5% w/v agar at 25 °C. Genomic DNA was extracted as described previously (van der Nest et al. 2021) and used to generate one paired-end library (550 bp insert size and read length of 251 bp) that was then sequenced using the Illumina HiSeq 2500 platform at Macrogen (Seoul, Korea). After duplicate and poor quality reads were removed using the Qiagen Genomics Workbench v. 20.0.4 (CLCBio, Aarhus), the remaining reads were assembled using SPAdes v. 3.13.0 (Bankevich et al. 2012). The completeness of the genome assembly was determined with BUSCO v. 4.0.6 utilising the “hypocreales” dataset (Manni et al. 2021). We used the MAKER annotation pipeline (Cantarel et al. 2008), which uses Augustus (Stanke et al. 2006), Genemark ES (Ter-Hovhannisyan et al. 2008) and SNAP (Korf 2004) to annotate the assembly. In these procedures, annotation data from *F. circinatum* (Wingfield et al. 2012), *F. fujikuroi* (Wiemann et al. 2013), *F. verticillioides* (Ma et al. 2010), *F. mangiferae* and *F. proliferatum* (Niehaus et al. 2017) were included as supporting evidence for gene models.

Placement of *F. marasasianum* CMW 25512 within the FFSC was verified using phylogenetic analysis of a dataset containing translation elongation factor 1-α and β-tubulin gene sequences for relevant FFSC taxa (Duong et al. 2021). For this purpose, sequences were aligned using MAFFT v. 7.487 (Katoh et al. 2019), concatenated and subjected to maximum likelihood phylogenetic analysis in PhyML v. 3.1 (Guindon et al. 2010). As indicated by jModelTest v. 2.1.10 (Darriba et al. 2012), the analysis employed the generalised time reversible (GTR) model (Tavare 1986) with a proportion of invariable sites and gamma correction for among site rate variation.

### Results and discussion

**Fig. 1:**
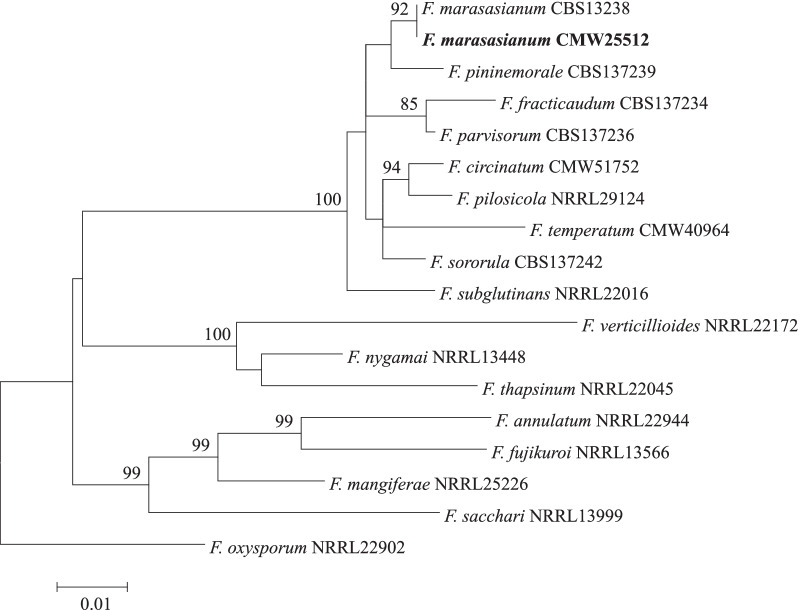
Maximum likelihood tree based on the partial gene sequences of translation elongation factor 1-α and β-tubulin (Duong et al. 2021; Herron et al. 2015; Wingfield et al. 2015a, 2018a). Values at branch nodes are the bootstrapping confidence values with those ≥ 85% shown. The *F. marasasianum* isolate sequenced in this study is indicated in bold; *F. marasasianum* CBS 137238 is ex-holotype

Assembly of the *F. marasasianum* genome yielded a total genome size of 47,207,981 bp with a G + C content of 46.25%. The assembly consisted of 166 contigs with a N50 of 1,535,275 bp, and phylogenetic analysis confirmed the taxonomic identity of the sequenced genome as *F. marasasianum* (Fig. 1). Genome completeness was estimated to be 99.8% corresponding to 99.6% complete and single-copy BUSCOs, 0.2% complete and duplicated BUSCOs and 0.2% missing BUSCOs (n = 4494). A total of 15,564 gene models were predicted in the *F. marasasianum* assembly with a density of 329.69 orfs/Mbp. Sequence analysis showed that the twelve chromosomes typically present in species from the FFSC are found in *F. marasasianum* CMW 25512.

In addition to *F. circinatum*, the genomic resource presented here represents the fifth genome (van der Nest et al. 2021; Wingfield et al. 2017, 2018b) for species associated with pitch canker-like symptoms on *Pinus* spp. (Herron et al. 2015). It has been suggested that these *Fusarium* species diversified alongside pines in Mexico/Central America (Herron et al. 2015; O’Donnell et al. 1998) and that their distribution is driven by international trade, thereby posing significant quarantine risks (Drenkhan et al. 2020). Availability of genomic resources for these fungi will facilitate and stimulate research aimed at resolving questions regarding their shared evolutionary history, ecology and pathogenicity.

*Authors:* Lieschen De Vos*, Tuan A. Duong, Magriet A. van der Nest, Emma T. Steenkamp, and Brenda D. Wingfield

*Contact: lieschen.bahlmann@fabi.up.ac.za

## IMA GENOME-F 16B

### Draft genome sequence of *Huntiella abstrusa*

#### Introduction

The family *Ceratocystidaceae* was redefined in 2014 to accommodate genera previously treated as species complexes in the genus *Ceratocystis s. lat.* (de Beer et al. 2014; Wingfield et al. 2013). One of these genera, *Huntiella*, now accommodates species previously placed in the *C. moniliformis* species complex and includes 31 species (Liu et al. 2018, 2020; Marin-Felix et al. 2019). Amongst the genera in *Ceratocystidaceae*, *Huntiella* species are defined by being typically saprobic, while many other species in this family are important plant pathogens or agents of blue stain (de Beer et al. 2014).

While most of the recognized *Huntiella* species are heterothallic, a small number have been described as unisexual (Wilson et al. 2015, 2021a). In heterothallic species such as *H. omanensis* and *H. bhutanensis*, individuals harbour either the *MAT1-1* or *MAT1-2* idiomorph, which confer the MAT1-1 and MAT1-2 mating types, respectively (Wilson et al. 2015, 2021a). Sexual development consequently requires an interaction between two individuals of opposite mating type, as in other heterothallic ascomycetes (Wilson et al. 2021b). In contrast, only MAT1-2 isolates of *H. moniliformis* and *H. fecunda* have been discovered and despite the absence of the MAT1-1 mating type, these species are capable of independent sexual reproduction via the unisexual pathway (Liu et al. 2018; Wilson et al. 2015).

In this study, we sequenced the genome of *Huntiella abstrusa*, using both the PacBio and Illumina platforms in an effort to generate a high-quality draft genome sequence. This species was described based on only a single isolate and was originally described as unisexual (Marin-Felix et al. 2019). The results from this study showed that the isolate that was used in the original description represented a mixed culture, with individuals of both the MAT1-1 and MAT1-2 mating types present. This species is thus heterothallic.

#### Sequenced strain

**Indonesia**: *Riau province*: Teso East, S 0° 04′ 33, E 101° 37′ 23, isolated from the bark of *Eucalyptus* spp. (*Myrtaceae*), 2005, *M. Tarigan* (PREM 61671—holotype; culture ex-type CBS 142243 = CMW 21092).

#### Nucleotide sequence accession number

This Whole Genome Shotgun project has been deposited at DDBJ/ENA/GenBank under the accession: JAJNMT000000000. The version described in this paper is version JAJNMT000000000.

#### Materials and methods

Genomic DNA was extracted from *H. abstrusa* using a rapid salt-extraction protocol (Aljanabi and Martinez 1997), with modification (Duong et al. 2013). For the long-read sequencing, a library was constructed from DNA extracted from a mixed mating type culture of *H. abstrusa* and sequencing was conducted at Macrogen (Seoul, Korea) using PacBio RSII 10 Kb SMRTbell template libraries and the DNA polymerase binding kit P6 v. 2. For the short-read sequencing, a library was constructed from DNA extracted from a single MAT1-2 individual that was isolated from the mixed mating type culture used for the long-read sequencing. An Illumina library was prepared using the TruSeq PCR free library kit with 550 bp median insert size and sequenced at Macrogen (Seoul, Korea) using the HiSeq 2500 platform, generating paired end reads of 251 bp.

The PacBio reads were assembled using Flye v. 2.8.1 (Kolmogorov et al. 2019). This assembly was subsequently polished with the trimmed Illumina reads using Pilon v. 1.23 (Walker et al. 2014). Three iterations of polishing were done to generate the final assembly. Genome statistics were summarized using Quast v. 5.1 (Gurevich et al. 2013). Genome completeness was evaluated using BUSCO v. 4.0.6, using the fungi_odb10, ascomycota_odb10, and sordariomycetes_odb10 lineage datasets (Simão et al. 2015). AUGUSTUS v. 3.2.3 was used to annotate protein coding genes, using the *Fusarium graminearum* gene models (Stanke et al. 2006).

Phylogenetic analyses were conducted to confirm the identity of the isolate used for genome sequencing. The sequences for three gene regions (ITS, BT1 and TEF-1α) were extracted from the sequenced genome and combined with homologous sequences from seven other *Huntiella* species (Liu et al. 2020). Each gene region was aligned independently using the online version of MAFFT v. 7.0 (Katoh and Standley 2013) with default settings. MrModelTest2 v. 2.4 (Nylander 2004) was used to conduct model testing on each alignment, after which the alignments were concatenated into a single file. MrBayes v. 3.2.7 was subsequently used for Bayesian inference analyses. This analysis was run for 500,000 generations, with 10 parallel runs, 4 chains, and using the models as identified by MrModelTest2. Trees were sampled every 100 generations and 25% of the sampled trees were discarded as burn-in. Posterior probabilities were calculated from the remaining trees.

#### Results and discussion

**Fig. 2:**
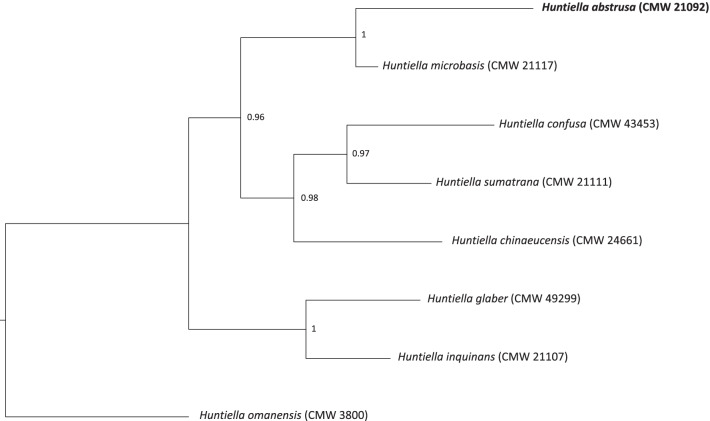
Identity confirmation of the *Huntiella abstrusa* isolate sequenced in this study. Three gene regions (ITS, BT1 and TEF-1α) were extracted from the assembled genome and compared to other species within the Asian clade of the genus *Huntiella*. The phylogeny was produced using Bayesian Inference and posterior probabilities are indicated at the nodes

The genome sequence of *H. abstrusa* was 29.5 Mb; assembled into a total of 287 contigs, 274 of which were above 1000 bp in length. The N50 and N90 values were 420,278bp and 67,803bp, respectively, and the L50 and L90 values were 18 and 76, respectively. The GC content was 48.5%. The BUSCO analysis showed that the assembly was 97.9%, 96.3%, and 86.9% complete with respect to the fungi, *Ascomycota*, and *Sordariomycete* datasets. A total of 7 952 protein coding genes were predicted by AUGUSTUS. The phylogenetic placement of this isolate was confirmed (Fig. 2), showing that *H. abstrusa* is most closely related to *H. microbasis* and others in the so-called Asian clade.

The results showed that the isolate used to describe *H. abstrusa* and used for the long-read genome sequencing in this study represented a mixed culture. This isolate consisted of individuals of both the MAT1-1 and MAT1-2 mating types. Assembly with the PacBio reads preferentially assembled the *MAT1-2* idiomorph due to low coverage of the *MAT1-1* idiomorph. Furthermore, the short-read genome sequencing was conducted using DNA from a single MAT1-2 isolate. Thus, the final genome, assembled using PacBio reads and polished used Illumina reads, represented a single MAT1-2 isolate. As expected, the *MAT* locus was associated with *SLA2* and *APC*, genes that are frequently present near the *MAT1* locus in *Pezizomycotina* species (Wilken et al. 2017). The *MAT1-2* idiomorph harboured the *MAT1-2-1* and *MAT1-2-7* genes, which is typical for *Huntiella* species (Wilson et al. 2015, 2021a) and other *Ceratocystidaceae* species (Krämer et al. 2021; Nel et al. 2018; Wilken et al. 2018).

The *H. abstrusa* genome was slightly larger than some of the other *Huntiella* genomes that have been sequenced to date (Table 1), including *H. moniliformis* (van der Nest et al. 2014b), but is smaller than that of *H. omanensis* (van der Nest et al. 2014a). This size difference can be accounted for by the difference in the number of genes in each of these genomes, with the average number of ORFs per Mb remaining stable across the species. Future research may focus on constructing the pangenome of these species, identifying the genes that are differentially present/absent in these species and determining whether their functions can be linked to their lifestyles.

**Table 1:** Genome statistics of the draft genome assemblies available for various *Huntiella* species

	Size (Mb)	No. of contigs	No. of genes	ORFs/Mb	References
*Huntiella abstrusa*	*29.5*	*274 (*> *1000 bp)*	*7952*	*270*	*Described here*
*Huntiella omanensis*	31.5	1638 (> 1000 bp)	8395	267	van der Nest et al. (2014a)
*Huntiella moniliformis*	25.4	365 (> 500 bp)	6832	269	van der Nest et al. (2014b)
*Huntiella bhutanensis*	26.8	448 (> 500 bp)	7261	271	Wingfield et al. (2016)
*Huntiella decipiens*	26.7	638 (> 500 bp)	7254	272	Wingfield et al. (2017)

The genome sequence of *H. abstrusa* presented here is the fifth to be published for a species of *Huntiella* (van der Nest et al. 2014a, b; Wingfield et al. 2016, 2017) and is one of more than 15 genomes for species in *Ceratocystidaceae* (van der Nest et al. 2014a; Wilken et al. 2013; Wingfield et al. 2016). Numerous *Huntiella* species have recently been the subject of investigations considering their mating behaviours and sexual strategies as well as the gene content and distribution of the *MAT* loci and pheromone response pathway (Wilson et al. 2018, 2020, 2021a). The availability of the genome of *H. abstrusa* will allow for further genome comparisons between species that exhibit different sexual strategies. Furthermore, there are notable differences between *Huntiella* species, which are almost exclusively saprobic, and species from other *Ceratocystidaceae* genera, which are typically pathogenic (de Beer et al. 2014). Thus, this genome and others like it will contribute towards a better understanding of the underlying genetic mechanisms that govern the ecology of these fungi, especially pathogenicity, virulence, and host specificity.

*Authors* ***:*** Andi M. Wilson*, Tuan A. Duong, Michael J. Wingfield and Brenda D. Wingfield

*Contact: andi.wilson@fabi.up.ac.za

## IMA GENOME-F 16C

### Draft genome sequences for two different isolates of the stem canker pathogen *Immersiporthe knoxdaviesiana*

#### Introduction

The *Cryphonectriaceae* include many important tree pathogens (Gryzenhout et al. 2009), notably the causal agent of the devastating chestnut blight, *Cryphonectria parasitica*. *Immersiporthe knoxdaviesiana* was first reported causing a serious stem canker disease on native *Rapanea melanophloeos* in a botanical garden in the Western Cape Province of South Africa (Chen et al. 2013). The disease was apparently new to the area and was observed to be spreading rapidly. Pathogenicity trials showed that *I. knoxdaviesiana* is aggressive on *R. melanophloeos* and able to kill trees in a short period of time (Chen et al. 2013). This has led to the suggestion that the fungus is an introduced pathogen (Wingfield et al. 2020). However, population genetic studies, for example utilizing microsatellite markers would be needed to confirm the hypothesis and thus clarify the origin of the pathogen.

Twelve genome sequences are currently available for species of *Cryphonectriaceae*. These include, *Chrysoporthe austroafricana* (Wingfield et al. 2015b), *C. cubensis*, *C. deuterocubensis* (Wingfield et al. 2015a), *C. puriensis* (van der Nest et al. 2021), *Celoporthe dispersa* (Liu et al. 2019), as well as seven species of *Cryphonectria* (https://www.ncbi.nlm.nih.gov/genome/). The aim of this study was to provide genome sequence data for *I. knoxdaviesiana*, a fourth genus of *Cryphonectriaceae*. This study also included a newly collected isolate of the pathogen from an area distant from where the canker disease was first recorded.

#### Sequenced strains

*Immersiporthe knoxdaviesiana*: **South Africa**: *Western Cape Province*: Harold Porter National Botanical Garden, *Rapanea melanophloeos*, 2011, *J. Roux, S.F. Chen, & F. Roets* (PREM 60740—dried culture; isolate CMW 37318 = CBS132864—ex-paratype culture). *Eastern Cape Province*: Haga Haga, *Rapenea melanophloeos*, 2019, *M.J. Wingfield* (CMW 55904 = CERC 8815). The latter isolate was the first to be collected from infected trees outside the area where the disease was first reported.

#### Nucleotide sequence accession number

The genomic sequences of *Immersiporthe knoxdaviesiana* (CMW 37318 and CMW 55904) have been deposited at DDBJ/EMBL/GenBank under the accession number ASM2111731v1 and JAJNGR000000000 respectively**.**

#### Material and methods

*Immersiporthe knoxdaviesiana* isolates CMW 37318 and CMW 55904 were obtained from the culture collection (CMW) of the Forestry and Agricultural Biotechnology Institute (FABI), the University of Pretoria and the latter culture has also been preserved in the collection of the China Eucalypt Research Centre (CERC), Chinese Academy of Forestry (CAF), Zhanjiang, Guangdong Province, China. Genomic DNA was extracted from single hyphal tip cultures grown on malt yeast broth (2% malt extract, 0.5% yeast extract) using the method described by Duong et al. (2013). To verify the identification of the isolates, the internal transcribed spacer (ITS) region and the partial β-tubulin gene (*tub1* and *tub2*) regions were sequenced. The reference sequences were obtained from GenBank and the sequence dataset was aligned using an online version of MAFFT v. 7 (Katoh and Standley 2013). Phylogenetic analysis using maximum likelihood (ML) was performed with RAxML v. 8 (Stamatakis 2014). Branch support was calculated using 1000 bootstrap replicates.

Nanopore sequencing was performed for the Western Cape isolate (CMW 37318) using the MinION sequencing device. The sequencing library was prepared using the Genomic DNA by Ligation (SQK-LSK109) protocol. The library was loaded on a MinION flow cell (R10.3) and the sequencing run was carried out for 48 h. Base calling was conducted using the ONT Guppy base calling software v. 4.0.14 (https://community.nanoporetech.com). Nanopore reads were trimmed using Porechop (https://github.com/rrwick/Porechop). The genome was assembled using Flye v. 2.7 (Kolmogorov et al. 2019) and polished using Rebaler v. 0.2.0 (Wick et al. 2019), which runs multiple rounds of Racon v. 1.4.13 (Vaser et al. 2017) followed by two rounds of polishing iterations with Medaka v. 1.0.3 (https://github.com/nanoporetech/medaka).

Illumina sequencing was performed for the Eastern Cape isolate CMW 55904. The genomic DNA was submitted to Novogene (Beijing, China) for sequencing using the Illumina HiSeq 2500 platform. A paired-end library with 550 bp median insert size was generated and 250 bp paired-end reads were sequenced. Poor quality data and adapters were removed using the program Trimmomatic v. 0.36 (Bolger et al. 2014). The program SPAdes v. 3.14 (Bankevich et al. 2012) was used to assemble the genome.

Protein coding gene models were annotated using AUGUSTUS v. 3.3.3 with *Fusarium graminearum* genemodels (Stanke and Morgenstern 2005). BUSCO v. 4.1.3 with the Sordariomycetes dataset (Simão et al. 2015) was used to evaluate completeness of the assembled genomes. The MAT1-1-1, MAT1-1-2, and MAT1-1-3 (AF380365; MAT1-1 accession) protein sequences of *C. parasitica* isolate EP44 and the MAT1-2-1 (AF380364; MAT1-2 accession) protein sequence of *C. parasitica* isolate Cr2b were used for local BLASTx searches applying a maximum e-value of 1E-10 with default settings to locate the putative *MAT* locus in the sequenced genomes.

#### Results and discussion

**Fig. 3:**
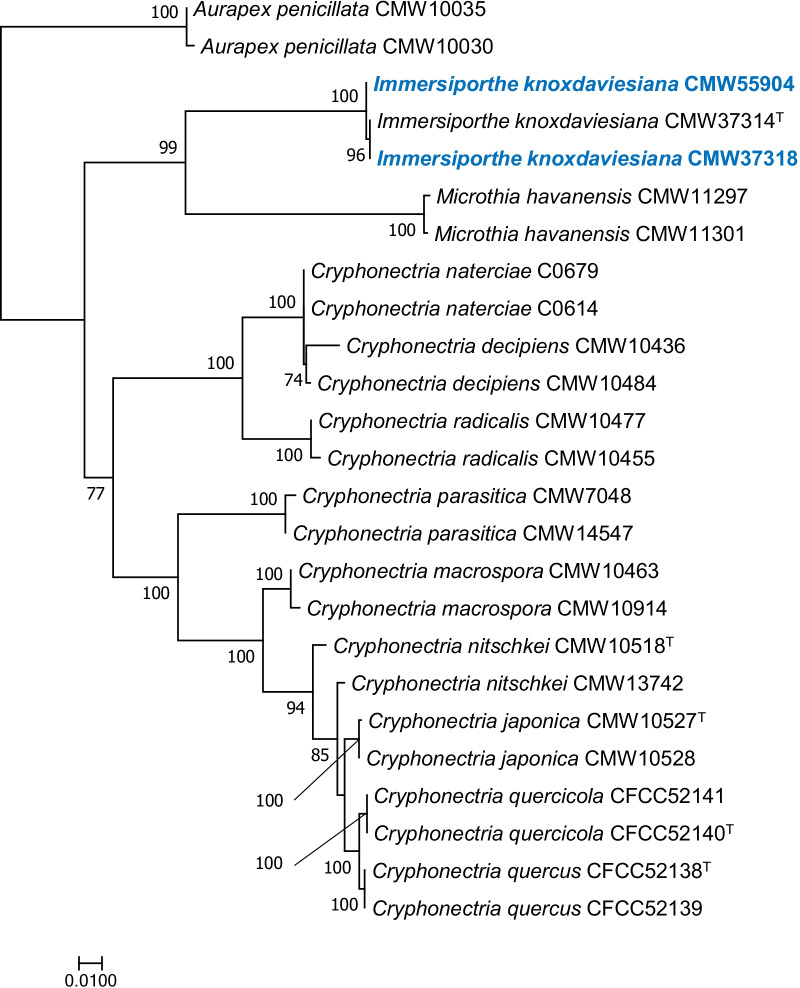
Maximum Likelihood tree based on ITS region and partial gene sequences of *but1* and *but2*. Bootstrap values > 70% are shown. The isolates used in this study are indicated in blue and bold. ^T^indicates ex-type isolate

Phylogenetic analysis confirmed the taxonomic identity of the two isolates as *I. knoxdaviesiana* (Fig. 3). The assembled genome size of the Western Cape isolate (CMW 37318) was 38,985 688 bp, with an N50 of 3,955,278 bp and L50 of 4, while the Eastern Cape isolate CMW 55904 had the assembled genome size of 39,205,829 with an N50 of 339,827 bp and L50 of 37. The assembly of the CMW 37318 isolate had 12 contigs and that of CMW 55904 had 421 contigs, of which 212 were longer than 1 Kb. The GC content was 53.9% for both isolates. AUGUSTUS predicted 11,116 and 10,984 protein coding gene models for isolates CMW 37318 and CMW 55904, respectively. BUSCO analysis showed the assembled genome of isolate CMW 37318 had an 87.9% completeness score. Of the 3817 BUSCO groups searched, 42 BUSCO orthologs were fragmented and 419 BUSCO orthologs were missing. Isolate CMW 55904 had a 98.3% BUSCO completeness score. Nine BUSCO orthologs were fragmented and 55 orthologs were missing.

**Fig. 4:**
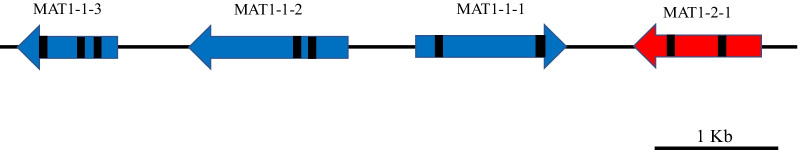
*MAT1* locus of *Immersiporthe knoxdaviesiana* predicted by AUGUSTUS v. 3.3.3. Predicted introns were denoted by black blocks

The results of BLASTx showed both *MAT1-1* and *MAT1-2* idiomorph were on the contig 4 of CMW 37318 and scaffold 39 of isolate CMW 55904, which suggests that *I. knoxdaviesiana* has a homothallic reproductive system (Fig. 4). The reference MAT1-1-1 protein sequence of *Cryphonectria parasitica* had a BLAST result of 327 bp with a pairwise identity of 62.6%. The reference MAT1-1-2 protein sequence had a BLAST result of 401 bp with a pairwise identity of 74.6%. The reference MAT1-1-3 protein sequence had a BLAST result of 140 bp with a pairwise identity of 73.6%. The reference MAT1-2-1 protein sequence had a BLAST result of 311 bp with a pairwise identity of 74.4%.

The estimated genome size and gene number for *I. knoxdaviesiana* is similar to those of other species in *Cryphonectriaceae*, such as *Chrysoporthe austroafricana* (44.6 Mb, 13,484 genes, Wingfield et al. 2015b), *C. cubensis* (42.6 Mb, 13,121 genes, Wingfield et al. 2015a), *C deuterocubensis* (43.9 Mb, 13,772 genes, Wingfield et al. 2015a), *C. puriensis* (44.7 Mb, 13,166 genes, van der Nest et al. 2021)*, Celoporthe dispersa* (40 Mb, 12,078 genes, Liu et al. 2019), and *Cryphonectria parasitica* (43.9 Mb, 11 184 genes, http://genome.jgi.doe.gov/Crypa2/Crypa2.home.html).

The genome sequences for the two *I. knoxdaviesiana* isolates from different locations elucidated in this study enable genomic comparisons between four distinct genera in *Cryphonectriaceae* as well as for numerous species in this important family. These genome sequences will aid in determining the origin of *I. knoxdaviesiana* and whether it could be an emerging alien invasive pathogen in South Africa.

***Authors:*** Hiroyuki Suzuki, FeiFei Liu, Tuan A. Duong*, ShuaiFei Chen, Michael J. Wingfield, and Brenda D. Wingfield

*Contact: tuan.duong@fabi.up.ac.za

## IMA GENOME-F 16D

### Draft genome assembly of *Macrophomina pseudophaseolina* strain WAC 2767, and ex-epitype strain of *M. phaseolina*

#### Introduction

The genus *Macrophomina* includes several economically important plant pathogenic species causing damping-off, seedling blight, and stem and dry root rot (aka charcoal rot) on a broad range of broadacre, horticultural, and vegetable crops worldwide (Kaur et al. 2012; Marquez et al. 2021). *Macrophomina* species are soil-borne pathogens and may survive in soil or plant debris for more than four years by forming resting structures called microsclerotia. Currently, five species of *Macrophomina* are known, viz*. M. phaseolina* and *M. pseudophaseolina* (Sarr et al. 2014), *M. euphorbiicola* (Machado et al. 2019), *M. vaccinii* (Zhao et al. 2019), and *M. tecta* (Poudel et al. 2021). Among these, *M. phaseolina* and *M. pseudophaseolina* (Sarr et al. 2014), have been reported on a broad range of host plants, with *M. phaseolina* alone reported on > 800 plant species (Farr and Rossman 2021; Sarr et al. 2014). However, the underlying molecular mechanisms allowing these two *Macrophomina* species to infect a wide range of plants is poorly understood.

In this study, we construct the draft genome assembly and annotation for a *M. pseudophaseolina* strain and provide genome assembly and annotation for the ex-epitype strain of *M. phaseolina*. These resources will facilitate comparative genomic studies of *Macrophomina* species to identify virulence-related factors, such as effectors and secondary metabolites, which will enhance future studies to better understand the evolution of *Macrophomina* species, host–pathogen interactions, and underlying infection mechanisms.

#### Sequenced strains

**Australia**: *Western Australia*: Kununurra, on *Arachis hypogaea*, Mar. 1980, *M.J. Barbetti* (WAC 2767).—**Italy**: *Siena*: on *Phaseolus vulgaris*, Sep. 1947, *G. Goidánich* (CBS 205.47—ex-epitype strain).

#### Nucleotide sequence accession numbers

The Whole Genome Shotgun project has been deposited at DDBJ/ENA/GenBank under the Accession Numbers JAJJIC000000000 and JAJJID000000000 (BioProject PRJNA780220; and BioSamples SAMN23133107 and SAMN23133108). The versions described in this paper are JAJJIC010000000 and JAJJID010000000.

#### Materials and methods

The *M. pseudophaseolina* strain (WAC 2767) sequenced here was obtained from the Western Australian Plant Pathology Reference Culture Collection (WAC, Perth, WA), and grown for 7 d in potato dextrose broth (Amyl Media, Australia) at room temperature (approx. 20 °C) at 220 rpm. Genomic DNA of strain WAC 2767 was extracted using a DNeasy Plant Mini Kit (Qiagen, Australia) according to manufacturer’s instructions. The DNA extracted from the ex-epitype strain of *M. phaseolina* (CBS 205.47) was obtained from the Westerdijk Fungal Biodiversity Institute in Utrecht, The Netherlands (formerly the CBS-KNAW Fungal Biodiversity Centre). DNA samples were quantified using a Qubit v.3.0 fluorometer (Thermo Fisher Scientific, Australia). Gel electrophoresis on a 0.8% agarose gel was used to assess DNA integrity.

**Fig. 5:**
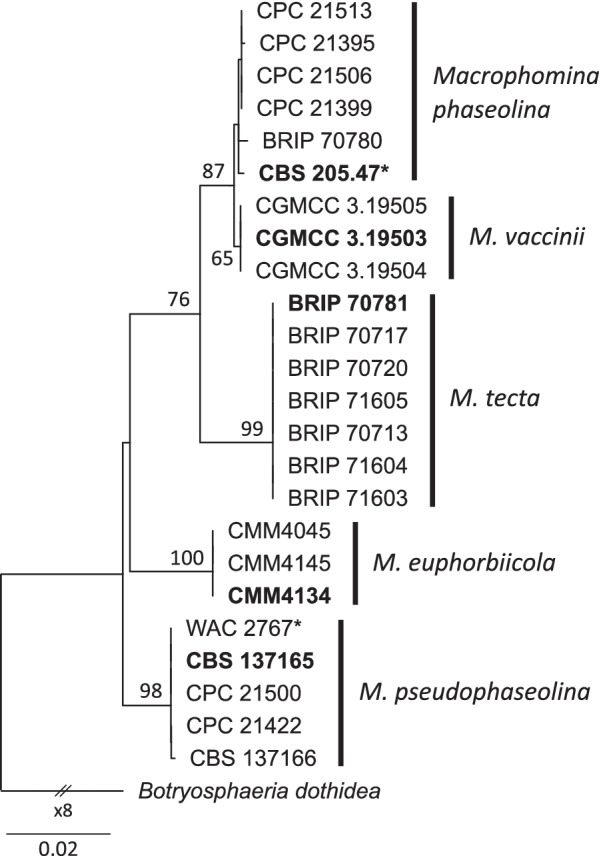
Maximum Likelihood phylogram of *Macrophomina* species inferred from the concatenated alignment of the internal transcribed spacer sequences of the nuclear ribosomal DNA and the intervening 5.8S region (ITS), actin, calmodulin, translation elongation factor one-alpha, and beta tubulin sequences obtained from Poudel et al. (2021) and Sarr et al. (2014). Alignment and tree were submitted to TreeBase (No. 28996). The tree was constructed using RAxML v.8 (Stamatakis 2014), based on the GTR substitution model with gamma-distribution rate variation for individual partitions. The tip labels in bold represent ex-type strains, and asterisks denote strains sequenced in the current study. Bootstrap support values > 80% are shown at the branches. The tree is rooted to *Botryosphaeria dothidea* (Poudel et al. 2021). The scale bar represents nucleotide substitutions per site

The identity of the strains as *M. pseudophaseolina* and *M. phaseolina* was confirmed through multi-locus phylogenetic analysis of five loci, namely, actin (*act*), calmodulin (*cmd*), internal transcribed spacers of the nrDNA and the intervening 5.8S region (ITS), translation elongation factor one-alpha (*tef1-α*), and beta tubulin (*tub2*) (Fig. 5). Sequence data for phylogenetic analysis were obtained from Poudel et al. (2021) and Sarr et al. (2014). Multiple sequence alignments were conducted in MAFFT v. 7.450 (Katoh and Standley 2013) and concatenated in Geneious Prime (Kearse et al. 2012, http://www.geneious.com). Maximum Likelihood phylogram of *Macrophomina* species was inferred from the concatenated alignment using RAxML v.8 (Stamatakis 2014), based on the GTR substitution model with gamma-distribution rate variation for individual partitions, and *Botryosphaeria dothidea* as the outgroup (Poudel et al. 2021).

Illumina libraries were prepared using Illumina’s Nextera™ DNA Flex library prep kit and Nextera™ DNA CD Indexes (Illumina, Singapore) according to the manufacturer’s protocol. A pooled library of CBS 205.47 and WAC 2767 was sequenced on an Illumina MiSeq platform using a 600-cycle paired-end reagents kits at the Agricultural Science and Engineering Precinct (ASEP) at the University of Southern Queensland, Australia.

The quality of the raw sequences was conducted using FastQC v.73 (Andrews 2010) in Galaxy portal (Afgan et al. 2018). Adapter and quality trimming of the raw reads was conducted using BBDuk from the BBmap suite v.38.90 (Bushnell 2014, settings: ktrim = r k = 23 mink = 11 hdist = 1 qtrim = rl trimq = 25 minlen = 45). The size of the genomes was estimated using Jellyfish v.2.3.0 (Marçais and Kingsford 2011). Genome assembly for each strain was performed using SOAPdenovo2 v.2.04 (Luo et al. 2012, kmers 21, 32, and 41) as well as Unicycler v. 0.4.8 (Wick et al. 2017) in Galaxy portal (Afgan et al. 2018). Unicycler produces short-read assemblies using SPAdes (Bankevich et al. 2012), followed by polishing using pilon v.1.23 (Walker et al. 2014). Assembly statistics for the final assemblies were estimated using QUAST v.5.0.2 (Gurevich et al. 2013). The completeness of the final assemblies was assessed using Benchmarking Universal Single-Copy Orthologs (BUSCO) v.5.1.2 (Simão et al. 2015) against the dothideomycetes_odb10 database (3786 core genes). Gene prediction was conducted in the genome annotation pipeline BRAKER2 v.2.1.6 (Hoff et al. 2019) using protein sequences of *M. phaseolina* strain 11–12 from strawberry (Burkhardt et al. 2019). This pipeline trained AUGUSTUS (Stanke et al. 2006) on the basis of spliced alignment information from protein of *M. phaseolina* strain from strawberry.

#### Results and discussion

Illumina paired end (2 × 300 bp) sequencing of *Macrophomina pseudophaseolina* strain WAC 2767 resulted in a total of 15,392,968 reads, with an estimated genome size of ~ 48.46 Mb based on Jellyfish analysis, which indicated a genome coverage of 96 ×. Unicycler-based assembly resulted in a higher quality genome based on the BUSCO result of 94.5% completeness (3,551 complete and single-copy BUSCOs, 27 complete and duplicated BUSCOs; 78 fragmented BUSCOs, 130 missing BUSCOs), and was selected for further analyses and annotation. The final assembly included 3799 contigs/scaffolds with an N50 value of 75.77 Kb and the largest contig of size 355.92 Kb. The GC content of the genome was 53.87%. BRAKER2 predicted 12,698 protein coding genes in the WAC 2767 genome.

Illumina sequencing of *M. phaseolina* strain CBS 205.47 resulted in a total of 27,631,588 reads. The estimated genome size based on Jellyfish was ~ 49.4 Mb, which is comparable to published genomes of *M. phaseolina* strains from jute, strawberry and sorghum (Burkhardt et al. 2019; Islam et al. 2012; Purushotham et al. 2020). An approximate genome coverage of 169 × was, therefore, achieved for strain CBS 205.47. Unicycler-based assembly produced a higher quality genome with 2407 contigs/scaffolds, N50 value of 66.80 Kb and the longest contig size of 320.52 kb. The GC content of the genome was 53.25%. BUSCO analyses (Dothideomycetes_odb10 database, 3,786 core genes) estimated 94.4% completeness (3,545 complete and single-copy BUSCOs, 30 complete and duplicated BUSCOs; 70 fragmented BUSCOs, 121 missing BUSCOs). BRAKER2 predicted 13,693 protein coding genes in the genome of strain CBS 205.47, which was comparable to the published *M. phaseolina* annotations (Burkhardt et al. 2019; Islam et al. 2012; Purushotham et al. 2020), ranging from 13,443 to 14,471 predicted proteins.

This is the first published genome for *M. pseudophaseolina* and will be valuable for future comparative studies of *Macrophomina* species. While multiple genome assemblies of *M. phaseolina* are publicly available, the genome sequence of the ex-epitype published here will serve as a reference for future phylogenetic and comparative genomics studies to further understand the biology and evolution of *M. phaseolina.*

***Authors:*** Barsha Poudel, Martin J. Barbetti, and Niloofar Vaghefi*

*Contact: niloofar.vaghefi@usq.edu.au

## IMA GENOME-F 16E

### Draft genome sequence of the basidiomycetous yeast *Naganishia randhawae* CBS 16859 isolated from avian guano in South Africa

#### Introduction

The genus *Naganishia* was initially proposed by Goto (1963) to accommodate the yeast *Naganishia globosus*. This species was subsequently subsumed into *Cryptococcus saitoi*, based on ribosomal RNA (rRNA) sequence analysis, which led to the synonymization of the genus (Fonseca et al. 2000). However, the genus was re-established with the purpose of resolving the diversity and heterogeneity of the yeast genus *Cryptococcus* (Fell et al. 1999; Fonseca et al. 2000; Liu et al. 2015; Scorzetti et al. 2002). *Naganishia* belongs to the class *Tremellomycetes*, order *Filobasidiales*, and family *Filobasidiaceae,* and comprises 16 species. Taxa in this genus reproduce by budding and sexual reproduction has to date not been observed (Kurtzman et al. 2011; Liu et al. 2015). They produce starch-like compounds and utilize caffeic, ferulic, hydroxybenzoic acids, L-malic, p-coumaric, protocatechuic, and vanillic acids (Fotedar et al. 2018; Liu et al. 2015). Nitrate is utilized and fermentation has not been observed (Liu et al. 2015).

Species in *Naganishia* have a global distribution particularly in extreme terrestrial environments characterized by cold temperatures and recurrent diurnal freeze/thaw cycles (Costello et al. 2009; Lynch et al. 2012; Pulschen et al. 2015; Schmidt et al. 2017; Solon et al. 2018). Several of these species are known to cause disease within immunocompromised individuals. *Naganishia albida* has been implicated in cutaneous lesions, encephalitis, keratitis, onychomycosis, and pneumonia (Burnik et al. 2007; Lee et al. 2004; Ragupathi and Reyna 2015); *N. diffluens* is associated with subcutaneous infections (Kantarcioǧlu et al. 2007); *N. friedmannii* has been confirmed as an etiologic agent of onychomycosis (Ekhtiari et al. 2017); and *N. uzbekistanensis* has been isolated from the bone-marrow of lymphoma patients (Powel et al. 2012).

Members of this genus also show great biotechnological value. *Naganishia liquefaciens* and *N. adeliensis* display an ability to accumulate lipids (Selvakumar et al. 2019; Selvakumar and Sivashanmugam 2018), while the draft genome of *N. albida* NRRLY-1402 incorporates several genes that play a role in lipid biosynthesis (Vajpeyi and Chandran 2016). Lipid production in yeast is considered an alternative feedstock for biodiesel production, thereby contributing to the fight against climate change and the development of sustainable practices (Luque et al. 2010). Similarly, the microbial production of single cell oils (SCOs) has received considerable attention in recent years. It provides a variety of advantages over the use of animal or plant sources, for example, it is not limited to climatic conditions or geographical location. Additionally, they also offer a shorter processing time and allow for a greater variety of substrate utilization, including industrial waste (Luque et al. 2010; Ward and Singh 2005).

Despite their potential economic and environmental importance, the mechanism underlying the diverse functions of *Naganishia* species is poorly understood. Here we report the first genome sequence of an isolate of *N. randhawae* CBS 16859, which was isolated from avian guano in South Africa. This genome will contribute towards an increased understanding of the biology of the genus *Naganishia*.

#### Sequenced strain

**South Africa**: *Gauteng*: Johannesburg, isolated from avian guano, 2018, identification confirmed *Kalonji A. Tshisekedi* (CBS16859).

#### Nucleotide sequence accession number

This Whole Genome Shotgun project and internal transcribed space sequence (ITS) of *Naganishia randhawae* CBS 16859 has been deposited at DDBJ/ENA/GenBank under the accession JABRPJ000000000 and MT542688 respectively.

#### Culture conditions

Our isolate differed from the described type material (CBS 10160; Khan et al 2010) in that melanin was produced on bird seed agar (BSA; Staib and Seeliger 1966) and growth was inhibited at 37 °C. *Naganishia randhawae* CBS 16859 was maintained by periodic transfer on yeast peptone dextrose (YPD, pH 5.5) agar (Ausubel et al. 1989) supplemented with 0.2 g/L of chloramphenicol (Sigma) and incubated at 30 °C.

#### Taxonomic placement

The taxonomic placement of *N. randhawae* CBS 16859 was investigated by constructing a Maximum Likelihood (ML) phylogeny with the fungal ITS 1 and 2 regions (including the 5.8S rRNA gene).

Genomic DNA was extracted using the Quick-DNA Fungal/Bacterial Kit (Zymo Research) as per the manufacturers’ instructions. The ITS region was amplified using the primers ITS1 and ITS4 and previously described protocol (White et al. 1990). The manually curated ITS sequence of *N. randhawae* CBS 16859 was deposited in GenBank (Accession No. MT5452688). Comparison of the amplified ITS nucleotide sequence against the National Center for Biotechnology Information (NCBI) nucleotide database indicated that this strain belongs to *N. randhawae*, sharing 99% nucleotide identity with the type isolate CBS 10160.

The ITS sequences of representative isolates in this genus were obtained from the NCBI nucleotide database (https://www.ncbi.nlm.nih.gov/nuccore). Similarly, the ITS sequence from *Filobasidium wieringae* CBS1937 (AF444373.1) was included as outgroup. All sequences were aligned using the M-Coffee webserver (Moretti et al. 2007), prior to trimming of the unaligned 5′ and 3′ ends. An ML phylogeny was then constructed using PhyML-SMS with smart model selection (Guindon et al. 2010) (Lefort et al. 2017) with 1000 bootstrap replicates.

#### DNA isolation, genome sequencing and assembly

Genomic DNA was extracted as previously described. Library preparation and sequencing were performed using the Illumina NovaSeq 6000 platform (paired-end read approach 2 X 250 bp) by MR DNA (Texas, USA). Adapter sequences and low quality (< Q28) reads were removed using the FastQC toolkit v. 0.11.8 (Andrews 2010). The trimmed reads were de novo assembled with SPAdes v. 3.9.0 (Bankevich et al. 2012). The assembled contigs were further refined with local Blastn analysis using BioEdit v.7.0.5.3 (Hall 1999) and contig extension using the Integrated Genome Browser v. 9.0.2 (Nicol et al. 2009).

#### Gene prediction and genome annotation

Genome completeness was evaluated with BUSCO v. 2.0 using the basidiomycota_odb9 reference dataset for benchmarking (Simão et al. 2015). The genome was ad functionally annotated using eggNOG-mapper v. 1.0.3.3-g3e22728, Interproscan v. 5.30–69.0, and PFAM v. 31.0 (Finn et al. 2014; Huerta-Cepas et al. 2019; Jones et al. 2014), according to the Clusters of Orthologous Groups of proteins (COGs). Putative enzymes involved in carbohydrate utilization were identified by searching against the Carbohydrate-Active enZymes databases (CAZymes, Lombard et al. 2014). Protease families were classified using the Basic Local Alignment Search (BLAST) tool against the MEROPS databases (Rawlings et al. 2016). Similarly, the genomes of *N. albida* JCM2334 (NCBI Acc. BCHV00000000.1) and *N. vishniacii* ANT03-052 (JGI Id 1001554) were annotated for comparative purposes.

#### Results and discussion

**Fig. 6:**
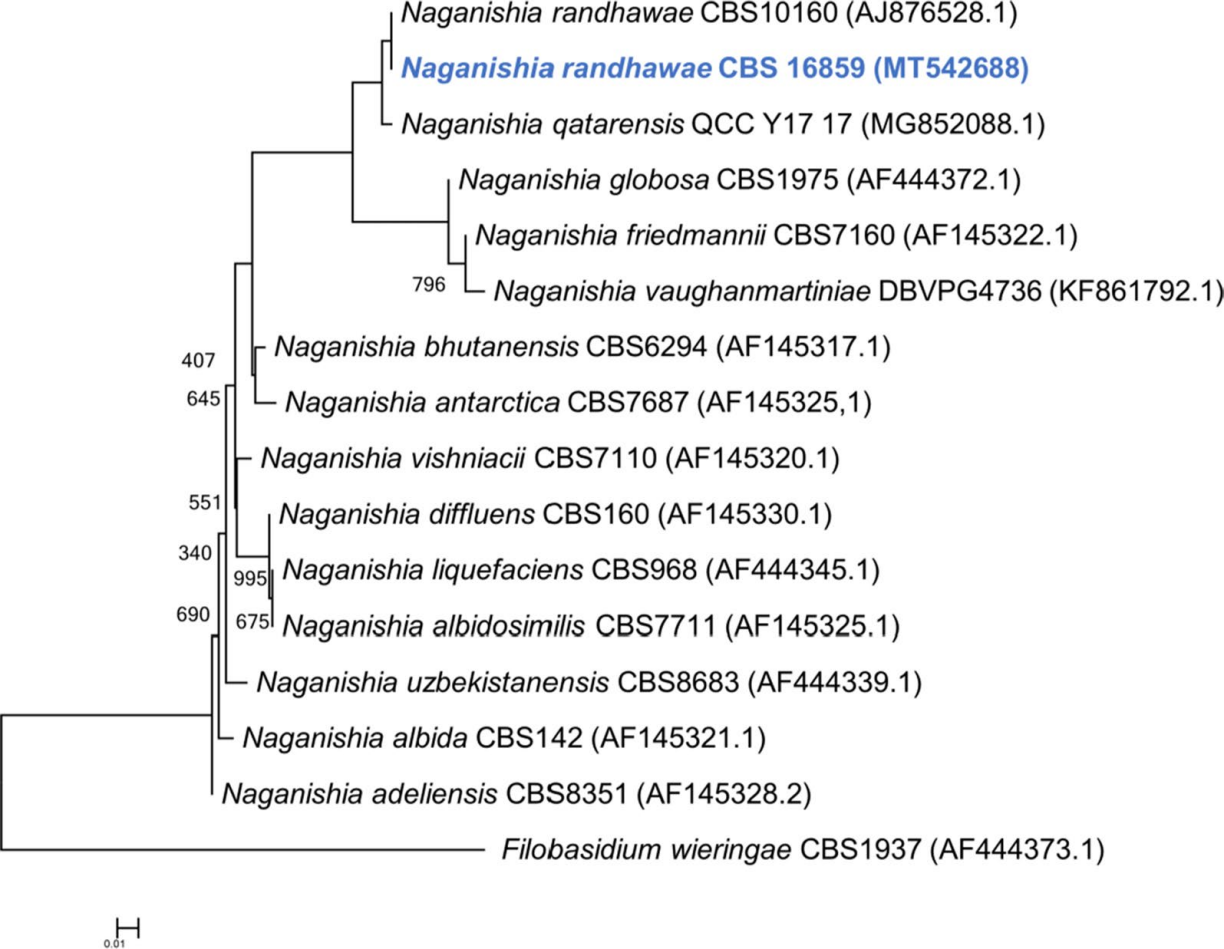
Maximum likelihood (ML) phylogeny indicating the taxonomic placement of *Naganishia randhawae* CBS 16859. The tree was constructed on the basis of the internal transcribed spacer region sequences using PhyML-SMS, with the best fit evolutionary model HKY85 + G (Guindon et al. 2010; Lefort et al. 2017). Bootstrap support values (*n* = 1000 replicates) greater than 500 are indicated at the nodes

The taxonomic placement of *N. randhawae* CBS 16859 within the genus *Naganishia* is illustrated in Fig. 6.

The draft genome sequence of *N. randhawae* CBS 16859 is comprised of 386 contigs with a total size of 20,271,596 bp and average G + C content of 51.71%. As such, it is approximately 0.58 Mb larger than the genome of *N. vishiniacii* ANT03-52 and 0.41 Mb smaller than that of *N. albida* JCM2334 (data not shown). The genome of *N. randhawae* CBS 16859 codes for 6,775 proteins and 168 rRNA sequences and incorporates 86.8% of the Basidiomycota BUSCO gene models (Simão et al. 2015), showing a relatively high level of completeness.

**Fig. 7:**
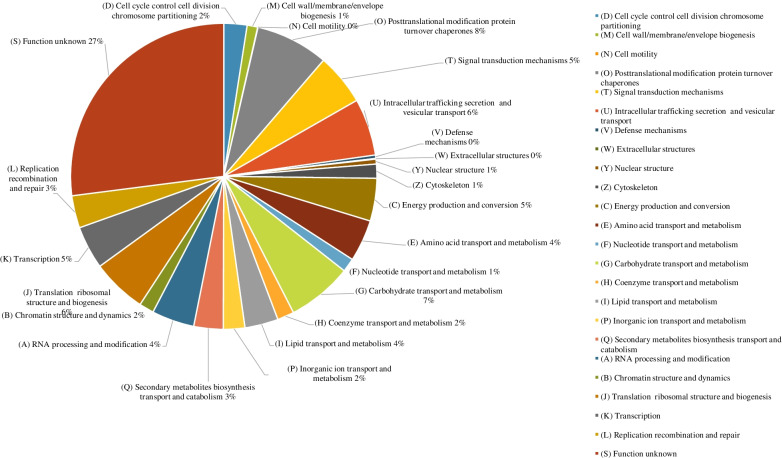
Clusters of Orthologous Groups (COGs) of functional categories and their relative abundances in the *Naganishia randhawae* CBS 16859 genome (Huerta-Cepas et al. 2019)

The genome of the *N. randhawae* CBS 16859 was annotated using eggNOG according to the COGs database (Huerta-Cepas et al. 2019). The predominant functional categories comprised of proteins with known functions included the “posttranslational modification protein turnover chaperones” (O) category with 8%; followed by the “carbohydrate transport and metabolism” (G) category with 7% (Fig. 7). Metabolic variation in yeast holds practical importance as it provides an insight regarding the growth rate, biotechnological importance, and level of pathogenicity of the species (Breunig et al. 2014; Gibney et al. 2013).

Carbohydrate-Active Enzymes (CAZymes) are responsible for the synthesis and degradation of glycoconjugates, oligo-and polysaccharides and are also active in immune and host-pathogenic interactions. Analysis of CAZymes showed that the largest number of genes in the auxiliary activity (AAs) family was encoded by *N. randhawae* CBS 16859 (Table 2, Lombard et al. 2014). The AAs family consists of multicopper oxidases (MCOs) that include some of the essential enzymes involved in melanin biosynthesis (Langfelder et al. 2003). Interestingly, the genome of *N. randhawae* CBS 16859 encodes a protein which is identical to the MCO laccase enzyme (KEP53184.1) found in the well-known melanin producing plant pathogen *Rhizoctonia solani* (Shu et al. 2019). Laccase activity and pigment has been shown to contribute towards pathogenesis in *N. albida* and *N. diffluens* (Ikeda et al. 2002), by inhibiting phagocytosis by macrophages, decreasing susceptibility to killing by free radicals and increasing resistance towards antifungal agents such as amphotericin B (Nosanchuk and Casadevall 2003). Similarly, the *N. randhawae* CBS 16859 genome also houses three unique copies of the ERG24 gene, which encodes the enzyme Delta-(14)-sterol reductase. Overexpression of this gene, and erg24 mutations, are associated with fungal resistance towards three classes of ergosterol inhibitors, specifically the allylamines, azoles and morpholines (Almeida-Paes et al. 2017; Li et al. 2016; van de Sande et al. 2007).

**Table 2:** Overview of CAZyme and number of gene families in each CAZyme category across the genomes of *Naganishia albida* JCM2334; *N. randhawae* CBS 16859 and *N. vishniacii*. ANTT03-052 (Lombard et al. 2014)

Category	*N. albida* JCM2334	*N. randhawae* CBS 16859	*N. vishniacii* ANT03-052
AAs	22	31	17
CBMs	6	5	8
CEs	18	17	14
GHs	221	228	238
GTs	45	43	42
PLs	4	3	3

**Fig. 8:**
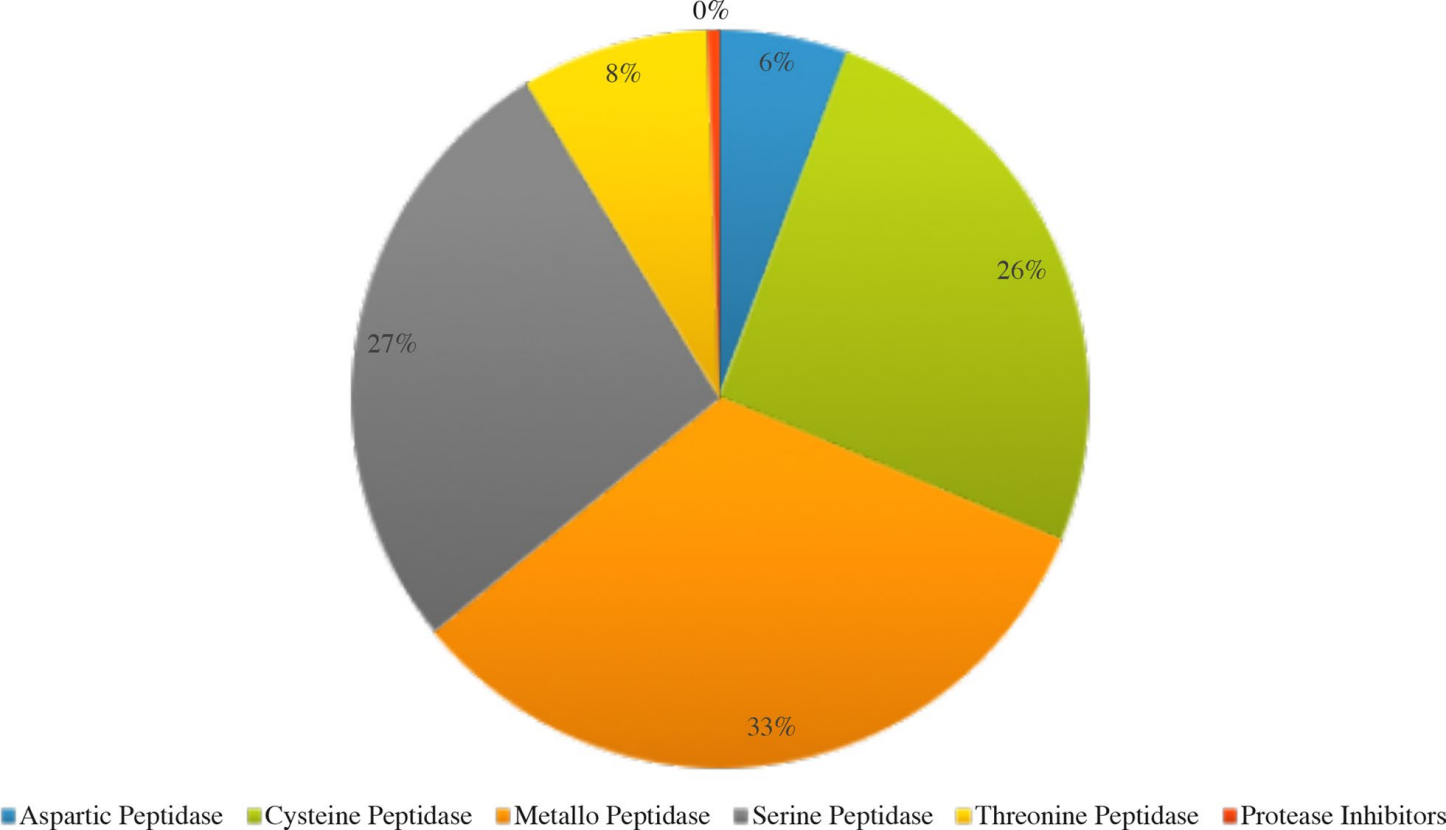
Classification of peptidases, according to the MEROPS database, encoded within the *Naganishia randhawae* CBS 16859 genome (Rawlings et al. 2016)

Coupled with these potential pathogenicity factors, analysis of the secreted proteases using the MEROPS database reveals that metallo- and serine proteases encoding genes were among the most abundant peptidase encoding genes in the genome of *N. randhawae* CBS 16859 (Fig. 8). Both enzyme families are important pathogenicity factors among *Naganishia* spp. and other fungal dermatophytes (Monod et al. 2002; Yike 2011).

Several genes coding for enzymes involved in lipid biosynthesis were also identified in the genome of *N. randhawae* CBS 16859, namely; acetyl-coenzyme-A carboxylase, fatty acid synthase, isocitrate dehydrogenase, malate dehydrogenase, malic enzyme, putative AMP deaminase, pyruvate decarboxylase, and pyruvate dehydrogenase (Adrio 2017; Ratledge 2004; Shen et al. 2016; Tang et al. 2009; Tehlivets et al. 2007). Such genes are also present in the genomes of *N. albida* JCM2334 and *N. vishiniacii* ANT03-52 and have been reported in other oleaginous yeasts, such as *Apiotrichum porosum* DSM 27194 (Gorte et al. 2019), *N. albida* SNRRL-Y-1402 (Vajpeyi and Chandran 2016) and *Trichosporon fermentans* CICC 1368 (Shen et al. 2016). These yeasts have shown great potential as lipid accumulators which could be used in the production of SCOs.

In conclusion, the genome sequence of *N. randhawae* CBS 16859 represents the first genome for this species and will serve as a valuable genomic resource to deepen our understanding of the biology, pathogenesis and biotechnological potential of the genus *Naganishia*.

***Authors:*** Kalonji A. Tshisekedi, Habibu Aliyu, Pieter de Maayer, and Angela Botes*

*Contact: angela.botes@wits.ac.za

## IMA GENOME-F 16F

### Draft genome sequence of *Pseudocercospora cruenta* causing black leaf mould of cowpea

#### Introduction

Cowpea (*Vigna unguiculata*) is a widely cultivated legume in tropical and subtropical regions, especially in Africa, Asia, and some parts of America. It serves as a significant source of carbohydrates, protein, minerals and vitamins for human and livestock nutrition in the tropical world (Duangsong et al. 2016; Longe 1980; Singh et al. 2003). In many countries, cowpea is grown as a major component in cropping systems because of its rapid growth, drought tolerance, and ability to fix atmospheric nitrogen (Duangsong et al. 2016; Omoigui et al. 2019). Cowpeas are planted on an estimated 14.5 mha of land per year, with a total yield of 6.2 million metric tons/year (Kebede and Bekeko 2020). India is one of the major countries contributing substantially to the cowpea production of the world. However, yields in India are significantly lower as compared to the world's average due to the unavailability of high yielding varieties and the occurrence of biotic and abiotic stresses (Raina et al. 2020).

Cowpea yield is not only lowered by the unavailability of high yielding varieties, but also by diseases caused by several pathogenic organisms, such as viruses, bacteria, and fungi. Fungal diseases are devastating for the growth, development, and yield of cowpea (Singh 2005). About 40 fungal species have been reported to cause diseases associated with different cowpea varieties (Bailey et al. 1990). One such disease is 'black leaf mould' of cowpea caused by *Pseudocercospora cruenta* (formerly *Cercospora cruenta*). *Pseudocercospora* belongs to *Mycosphaerellaceae* (*Capnodiales*, *Dothideomycetes*), and several species have *Mycosphaerella*-like sexual morphs (Crous et al. 2013; Hyde et al. 2013; Kirk et al. 2013). It is a cosmopolitan genus of phytopathogenic fungi associated with many plant species, including several economically relevant hosts (Bakhshi et al. 2014; Crous et al. 2013). *P. cruenta* represents a distinct pathogen specific to *Vigna* and *Phaseolus* species (Crous and Braun 2003; Hsieh and Goh 1990). Those include *Phaseolus lunatus, P. vulgaris, Vigna mungo, V. sesquipedalis, V. sinensis,* and *V. sinensis var. catjang* (https://www.mycobank.org/). In India, *P. cruenta* was reported on *Dolichos, Phaseolus, Macrotyloma* and *Vigna* species (Kamal 2010).

Black leaf mould of cowpea is prevalent in the rainy season during high moisture and warm temperatures (Heng et al. 2020). In India, the disease is so severe in September–October that farmers cannot harvest a single pod due to complete defoliation within few days of infection (Pandey 2002). The disease causes 35 to 40% yield loss in susceptible varieties (Fery et al. 1976; Schneider et al. 1976). In addition, black leaf mould incidence on cowpea limits the leaf area available for photosynthesis resulting in reduced yield (Booker and Umaharan 2007; Ekhuemelo et al. 2019). Therefore, there is a need to understand the genome organization of *Pseudocercospora cruenta* that could assist in identifying the virulence gene(s) that control the disease.

#### Sequenced strain

**India**: Varanasi, Banaras Hindu University agricultural farm, isolated from infected leaf samples of *Vigna unguiculata*, Sept. 2009, *R. Chand* (Pscow-1; MCC 9095).

#### Nucleotide sequence accession numbers

The genome sequence of *P. cruenta* has been deposited in DDBJ/ENA/GenBank databases under the accession number JAASFE000000000; Bioproject PRJNA613165; Biosample SAMN14395397. The version described in this paper is version JAASFE010000000. The raw Illumina HiSeq sequence reads are deposited in NCBI-Sequence Read Archives (SRA) under accession SRX7980600. The genome annotation and data on predicted genes and effectors have been deposited in Mendeley data with DOI number 10.17632/g39mv8yp87.1.

#### Materials and methods

##### Identification, isolation and DNA extraction

*Pseudocercospora cruenta* infected cowpea leaves were collected from Banaras Hindu University agricultural farm, Varanasi, India, in 2009. Typical symptoms present on the leaves were observed, photographed (Nikon D5200, Nikon, Japan), and microscopic examination was carried out by scrapping the growth of the pathogen from the infected spots. Photographs of conidia and conidiophores were taken at 20 × and 40 × resolution using NIS-Elements imaging software. The pathogen was identified by comparing the microscopic characteristics of conidia and conidiophores with the MycoBank database. *P. cruenta* was isolated aseptically on Potato Dextrose Agar (PDA) medium, and colony characters were studied. *P. cruenta* was submitted to an International Depositary Authority (IDA) recognized repository, the National Centre for Microbial Resource, National Centre for Cell Science (NCMR-NCCS), Pune, India, with an accession number MCC 9095.

Liquid culture of the monoconidial isolate was grown in 30 ml Potato Dextrose Broth (PDB) medium and incubated for 7 d at 25 ± 1 °C. Fungal mycelium was harvested aseptically after 7 d, and genomic DNA was extracted using modified Cetyl Trimethyl Ammonium Bromide (CTAB) extraction protocol (Murray and Thompson 1980). Quantification of DNA was carried out using Eppendorf BioPhotometer®D30. The amplified products were visualized on 1.5% agarose gel.

##### Genome sequencing, assembly, and annotation

The library was prepared for sequencing on a HiSeq 2500 (Illumina) using 2 × 100 bp and 2 × 250 bp paired-end chemistry at the AgriGenome Labs (Kochi, India). Raw paired-end reads were quality-checked using FastQC (Andrews 2010). Adapters and low-quality reads with an average quality score of less than 30 were removed using AdapterRemoval v. 2.3.1 (Schubert et al. 2016). FastUniq v. 1.1 (Xu et al. 2012) was used to remove duplicates in paired short reads. Velvet v. 1.2.10 was used for de novo assembly (Zerbino and Birney 2008). A range of k-mers from 31 to 95 was used for Velvet assembly. Quality assessment of complete assembly statistics was performed in QUAST v. 4.6 (Gurevich et al. 2013). The quality and completeness of the assembly was assessed with Benchmarking Universal Single Copy Orthologs (BUSCO v. 2.0) using the ascomycete odb_9 dataset (Simão et al. 2015). AUGUSTUS (Stanke and Morgenstern 2005) was used to predict protein-coding genes from the assembled genome. Using the BLASTX v. 2.6.0 tool (https://blast.ncbi.nlm.nih.gov/) and an E-value cut-off of 10^–3^, the predicted gene functions were compared with the UniProt (The UniProt Consortium 2021) and the NCBI databases. The best BLASTX hit for each gene was chosen based on query coverage, identity, similarity score, and gene description. The anticipated genes were annotated in terms of molecular activities, cellular components, and biological processes using the UniProt and NCBI databases for gene ontology. The protein dataset was subjected to the CUPP webserver (Barrett and Lange 2019) to predict and classify Carbohydrate Active Enzymes (CAZymes).

##### Effector prediction and annotation

To identify putative effector genes, the following pipeline was used: The predicted proteome was analyzed using SignalP v. 5.0 (Armenteros et al. 2019) to filter non-secretory proteins. Secretory protein dataset was then subjected to TMHMM v. 2.0 (Krogh et al. 2001) to eliminate proteins with one or more transmembrane helices, followed by PredGPI (Pierleoni et al. 2008) to exclude glycophosphatidylinositol (GPI) anchored proteins that likely represent surface proteins rather than secreted effectors. WoLF PSORT (Horton et al. 2007) and DeepLoc v. 1.0 (Armenteros et al. 2017) were used to eliminate proteins destined to organelles. Proteins rich in cysteine content (≥ 4 cysteine residues) were identified and were subjected to EffectorP v. 2.0 tool (Sperschneider et al. 2018) to identify the potential effectors. The predicted effectors were functionally annotated using OmicsBox (Götz et al. 2008).

##### Phylogenetic analysis

The phylogenetic tree was constructed using the ITS region sequence extracted from the assembled genome sequence. The ITS region sequences of ex-type and reliably named strains, and sequences from Bakhshi et al. (2014) and Crous et al. (2013) were acquired from GenBank based on the closest similarity of the BLASTn search. MAFFT v. 7.0 (Katoh and Standley 2013) was used to align the sequences. MEGA v. 7.0 (Kumar et al. 2016) was used for the phylogenetic study. During the sequence alignment, gaps and missing data were removed. The phylogenetic tree was built using the Neighbour-Joining method (Saitou and Nei 1978). Bootstrap analysis was performed using 1000 repetitions to calculate the confidence levels for each branch. Bootstrap values less than 50% were not considered.

#### Results

##### Morphological and molecular identification

**Fig. 9:**
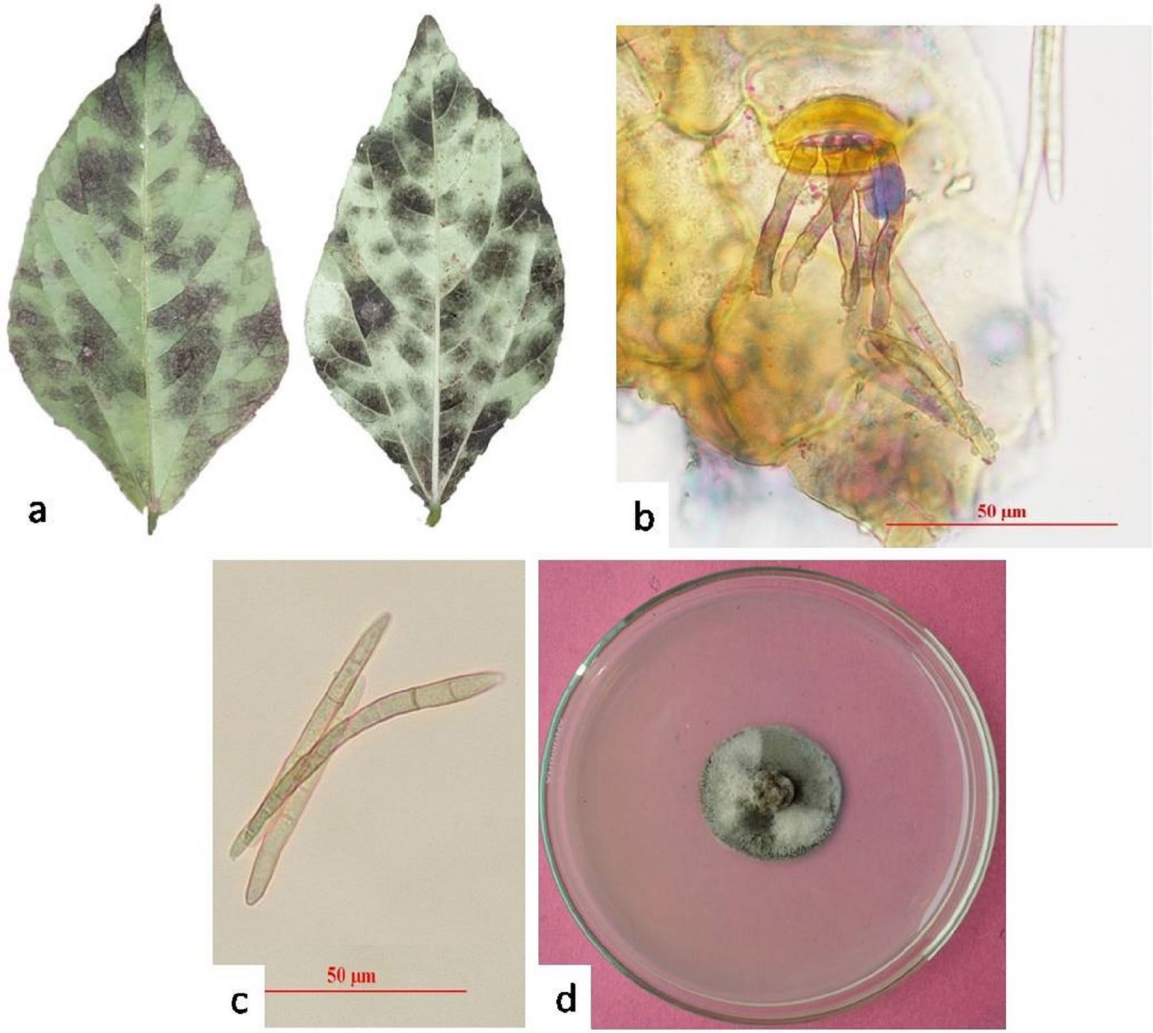
*Pseudocercospora cruenta* (isolate Pscow-1; MCC 9095) on *Vigna unguiculata* a-d, **a** Symptoms on adaxial and abaxial surface of leaf, **b** Conidiophore, **c** Conidia, **d** Colony on Potato Dextrose Agar (PDA) medium. Bars b and c = 50 μm

**Fig. 10:**
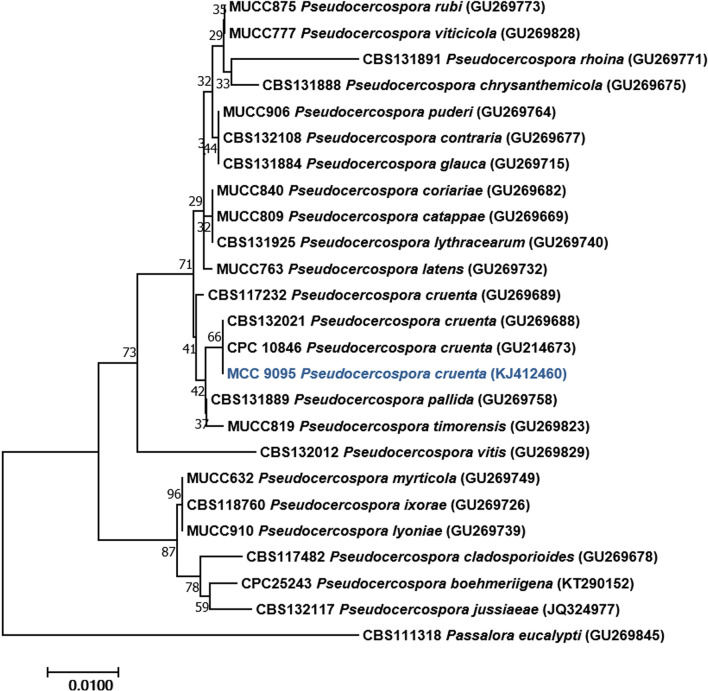
Neighbor-Joining (NJ) tree based on nrITS sequences to identify the sequenced *Pseudocercospora cruenta* (shown in bold). Sequence alignments were produced using MAFFT v. 7.0 and the phylogenetic tree was constructed using MEGA v. 7.0. The tree was rooted to *Passalora eucalypti*. Bootstrap support values from 1000 replicates are shown at the nodes. Bar = 0.01 nucleotide substitutions per site

The black leaf moulds were rusty brown to reddish, sometimes almost grey, and orbicular in shape. Symptoms were produced initially on the abaxial surface of older leaves. Mature leaves developed grey symptoms due to the superficial growth of the pathogen and its dark conidia (Fig. 9a). *P. cruenta* was characterized by the presence of rudimentary stromata, subhyaline to pale olivaceous brown conidiophores with 0–3 septa (Fig. 9b). Conidia were cylindric or cylindro-obclavate, subhyaline to very pale olivaceous brown, straight to mildly curved, with 3–14 septa (Fig. 9c). The cultured colony appeared dark grey to black on the PDA medium (Fig. 9d). The phylogenetic interference based on the ITS region confirmed the sequenced draft genome as the *P. cruenta* (Fig. 10).

##### Genome assembly and annotation

The draft genome assembly of *P. cruenta* resulted in a genome of 40.39 Mb with an overall GC content of 46.6%. There were 241 contigs larger than 50 Kb. Statistics of genome sequencing and assembly are summarised in Table 3. Gene prediction analysis yielded a total of 12,606 protein-coding genes. The number of predicted genes with a significant BLASTX match (E-value <  = 1e−3 and similarity score >  = 40%) with UniProt was 11,741. Genome completeness analysis identified 284/290 (97.93%) complete BUSCOs in the database of ascomycetes. Detailed characterization of the draft genome suggested that 502 genes were predicted to encode CAZymes, including 82 enzymes involved in auxiliary activities, 13 carbohydrate esterases, 311 glycoside hydrolases, 89 glycosyl transferases, and 7 polysaccharide lyases.

**Table 3:** Genome sequencing and assembly statistics of *P. cruenta*

Attributes	Statistics
Estimated genome size (bp)	4,03,94,060
Number of contigs (> = 50,000 bp)	241
Largest contig (bp)	9,10,576
GC (%)	46.6
N50	2,21,263
L50	70
Predicted protein-coding genes	12,606
Genes assigned to GO terms	2,325
Complete BUSCOs (C)	284
Complete and single-copy BUSCOs (S)	283
Complete and duplicated BUSCOs (D)	1
Fragmented BUSCOs (F)	1
Missing BUSCOs (M)	5
Total BUSCO groups searched	290

Gene ontology analysis mapped 1038 terms associated with molecular functions such as ATP binding, oxidoreductase activity, metal ion binding and nucleic acid-binding; 339 terms associated with cellular components where the maximum numbers of hits were linked to integral membrane transport and 948 terms related to biological processes such as transmembrane transport, metabolic processes, DNA repair and transcription.

##### Prediction and annotation of effectors

The genome of *P. cruenta* consisted of 12,606 predicted proteins. Out of those proteins, 818 were classically secreted proteins with a signal peptide. Out of 818 proteins, 86 proteins with one or more transmembrane helices and 68 proteins with GPI anchored motifs were eliminated. Out of the 664 proteins, WoLF PSORT and DeepLoc identified 456 extracellular and cytoplasmic proteins, out of which 93 proteins were predicted to be effector.

Functional annotation of the predicted effectors identified 44 (47%) proteins with hypothetical function. The remaining proteins identified were associated with biochemical functions like degradation of large molecules like hydrolases and peptidases, metabolic processes, metal ion binding, transcriptional co-activation, biosynthetic processes, necrosis induction, and proteolysis.

#### Discussion

*Pseudocercospora cruenta* has a broad host range resulting in infections being carried over to the next growing season (Omoigui et al. 2019). Therefore, black leaf mould management is difficult due to inoculum presence from multiple hosts. Furthermore, continuous use of fungicides for pathogen management causes harmful effects to the environment, degrades the beneficial soil microbiota, and increases production costs. Thus, there is a need to develop cost-effective and environment-friendly approaches such as developing black mould-resistant varieties to manage the disease. Therefore, this study was conducted to understand the *P. cruenta* genome organization. The estimated genome size of *P. cruenta* (40.39 Mb) is comparable to that of other *Pseudocercospora* species, including *P. pini-densiflorae* (43.51 Mb; https://www.ncbi.nlm.nih.gov/genome/30746), *P. eumusae* (47.12 Mb; Chang et al. 2016), and *P. macadamiae* (40.07 Mb; Akinsanmi and Carvalhais 2020). Wijayawardene et al. (2017) has accepted 1500 species under the genus *Pseudocercospora*. However, currently there are only eight genome assemblies available in the public databases.

Pathogenic fungi secrete key pathogenicity molecules referred to as effectors, which manipulate the host cell physiology to obtain nutrients, suppress plant defense and ultimately promote infection (Raffaele and Kamoun 2012). Effectors are small, cysteine-rich proteins with a predicted secretion signal discovered in plant pathogenic fungi. Understanding the genome organisation and identification of effectors of *P. cruenta* will serve as an important resource for future functional genomics studies. The computationally predicted effectors can be verified for further studies. Furthermore, this information can be compared with other genome assemblies available for better understanding of host–pathogen interaction and development of disease management strategies.

***Authors:*** Shagun Sinha, Sudhir Navathe, Ravindra N. Kharwar*, and Ramesh Chand*

*Contact: rc_vns@yahoo.co.in, rnkharwarbot@bhu.ac.in
